# The Role of RFamide-Related Peptide-3 in Age-Related Reproductive Decline in Female Rats

**DOI:** 10.3389/fendo.2016.00071

**Published:** 2016-06-21

**Authors:** Anna C. Geraghty, Sandra E. Muroy, Lance J. Kriegsfeld, George E. Bentley, Daniela Kaufer

**Affiliations:** ^1^Department of Integrative Biology, University of California Berkeley, Berkeley, CA, USA; ^2^Department of Psychology, University of California Berkeley, Berkeley, CA, USA; ^3^Helen Wills Neuroscience Institute, University of California Berkeley, Berkeley, CA, USA; ^4^Program in Child and Brain Development Toronto, Canadian Institute for Advanced Research (CIFAR), Toronto, ON, Canada

**Keywords:** GnIH, RFRP3, GnIH/RFRP3, aging, reproductive senescence

## Abstract

Reproductive senescence, the point in time when females cease to show estrous cyclicity, is associated with endocrine changes in the hypothalamus, pituitary, and gonads. However, the mechanisms triggering this transition are not well understood. To gain a better understanding of the top-down control of the transition from reproductive competence to a state of reproductive senescence, we investigated middle-aged female rats exhibiting varying degrees of reproductive decline, including individuals with normal cycles, irregular cycles, and complete cessation of cycles. We identified hormonal changes in the brain that manifest before ovarian cycles exhibit any deterioration. We found that females exhibit an increase in RFamide-related peptide-3 (RFRP3) mRNA expression in the hypothalamus in middle age prior to changes in estrous cycle length. This increase is transient and followed by subsequent decreases in kisspeptin (*KiSS1*) and gonadotropin-releasing hormone (*GnRH*) mRNA expression. Expression of *RFRP3* and its receptor also increased locally in the ovaries with advancing age. While it is well known that aging is associated with decreased GnRH release and downstream disruption of the hypothalamic–pituitary–gonadal (HPG) axis, herein, we provide evidence that reproductive senescence is likely triggered by alterations in a network of regulatory neuropeptides upstream of the GnRH system.

## Introduction

Female reproduction is a complex process that requires precise neurochemical timing. From puberty through senescence, a complex interplay among central neuroendocrine circuits, peripheral hormonal systems, and internal and external cues is required for successful procreation. The hormonal regulation that is involved in successful reproductive functioning is well studied. However, less is known about the neural and hormonal mechanisms initiating the degradation of the reproductive axis as females approach reproductive senescence.

Reproductive senescence in females is defined as the point in an organism’s life when it ceases to show regular cyclic ovarian activity, exiting from an active reproductive stage to an inactive reproductive life, akin to menopause in humans. In humans, menopause is characterized as three stages – premenopausal, when females still maintain regular menstrual cycles; perimenopausal, when cycles become increasingly irregular; and postmenopausal, defined as 1 year post amenorrhea (1) – and is associated with specific endocrine changes in both the brain and the ovaries (2–4). Rats represent a useful, non-human model for the study of menopause as they experience reproductive decline analogous to that of humans. With advancing age, middle-aged rats transition from regular 4- to 5-day estrous cycles to irregular cycle lengths of over 6 days, followed by a complete cessation of cyclicity, characterized as either a state of persistent estrus or diestrus. This final transition occurs in female rats between 12 and 15 months of age (5), appropriately corresponding to about 45–50 years old in women (4). The hypothalamic–pituitary–gonadal (HPG) axis is highly conserved across mammals, and the hypothalamic and ovarian changes in the rodent resemble menopausal changes in humans (6).

In female rodents, ovulation requires the precisely timed release of gonadotropin-releasing hormone (GnRH) from the hypothalamus. In rats, the transition to reproductive senescence is associated with reductions in GnRH neuron activity (7, 8) and secretion (9, 10) on the day of the preovulatory luteinizing hormone (LH) surge, resulting in an attenuation of LH and ovulation (10, 11). These changes are likely due to a modification in the network of upstream hypothalamic mediators of GnRH function. In particular, two neuropeptides identified in the past 15 years have been shown to regulate GnRH in opposing ways. Kisspeptin (the product of the *KiSS1* gene) found in the anteroventral periventricular (AVPV) and arcuate nuclei stimulates GnRH release, whereas RFamide-related peptide-3 (RFRP3) found in the dorsal medial hypothalamus of rodents acts to inhibit GnRH release (12–14). Kisspeptin mRNA levels decrease with aging (15), along with a decrease in the number of immunoreactive kisspeptin neurons in the AVPV in middle age (16). In contrast, the role of RFRP3 in female reproductive aging is yet to be discovered.

In this study, we examined *RFRP3* expression in regularly cycling young (3 months old), regularly cycling middle aged (8 months old), irregularly cycling (10 months old), and acyclic (12 months old) Long–Evans female rats. To explore the possibility that alterations in neurochemical systems upstream of GnRH neurons initiate the transition to reproductive senescence, we measured *RFRP3*; its receptors *GPR147*, *KiSS1*, and *GnRH*; and pituitary gonadotropin mRNA expression throughout aging. We found that *RFRP3* expression increased in middle-aged animals along with reductions in *KiSS1* and *GnRH* expression with advancing age. Expression of *RFRP3* and *GPR147* also increased with age in the ovaries. These data reveal an association between alterations in the RFRP3 and kisspeptin systems with age-related reproductive decline both centrally and peripherally.

## Materials and Methods

### Experimental Subjects

Adult female Long–Evans rats were housed in trios and exposed to a 12/12-h light–dark cycle. Lights came on at 0700 hours and *ad libitum* food and water were available. For all studies, rats were acclimated to their housing conditions for a week, and then vaginal smears were obtained daily to determine cyclicity for 30 days before the studies commenced. All tissue was collected between the hours of 0900 and 1200 hours. In the irregularly cycling group, only females that showed irregular cycles for at least 15 of the 30 days (cycles above 6 days in length) were used in the 10-month-old group (*n* = 21 of a total of 30 animals measured). For the acyclic group, only 12-month-old animals that exhibited persistent estrus or diestrus for at least 15 of the 30 days were used (*n* = 12 of a total of 18 animals measured). “Young” control animals were measured in the diestrus phase of the estrous cycle. All animal care and procedures were approved by the University of California Berkeley Animal Care and Use Committee.

### Real-time Reverse Transcriptase PCR

Rats were lightly anesthetized with isoflurane and rapidly decapitated before bilateral hypothalami (dissected from bregma −2.5 to bregma −3.5), pituitary, and whole ovaries were dissected on ice in RNA-later (Thermo Fisher Scientific, Santa Clara, CA, USA) and flash-frozen in liquid nitrogen. For all studies, total RNA was extracted using Trizol extraction methods and purified with DNase (DNA-free, Ambion). cDNA was synthesized following the manufacturer’s instructions for iScript cDNA synthesis kit (BioRad), and then RT-PCR was run using the manufacturer’s instructions for SsoAdvanced SYBR supermix (BioRad). Samples were run in a BioRad CFX96 real-time PCR system. After the PCR was complete, specificity of each primer pair was confirmed using melt curve analysis, and samples run on a 2% ethidium bromide agarose gel with a 50-bp DNA ladder (Invitrogen) to verify the generation of a single product of correct size. Rat primers were designed using the NCBI Primer BLAST software, which verifies specificity. The primer efficiencies were determined by standard curve, and *C*_t_ values were determined using PCR miner (17) and normalized to the ribosomal reference gene, ribosomal protein L16P (RPLP). There were no significant differences in RPLP values across any groups. Fold change was evaluated using the delta delta *C*_T_ method as outlined in Pfaffl 2001 (18).

Primer sequences are given below.

**Table:** 

Primer	Forward	Reverse	Temp	Product size
RPLP	ATCTACTCCG CCCTCATCCT	GCAGATGAGG CTTCCAATGT	55	159
RFRP3	CCAAAGGTTT GGGAGAACAA	GGGTCATGGC ATAGAGCAAT	55	110
GPR147	GGTCAGAACG GGAGTGATGT	AGGAAGATG AGCACGTAGGC	55	119
LHβ	GCAAAAGCCA GGTCAGGGATAG	AGGCCCAC ACCACACTTGG	55	92
FSHβ	TTCAGCTTTCCC CAGGAGAGATAG	ATCTTATGGTCT CGTACACCAGCT	55	305
Cga	CTATCAGTGTATG GGCTGTTG	CTTGTGGTAG TAACAAGTGC	55	199
KISS1	TGGCACCTGTG GTGAACCCTG	ATCAGGCGAC TGCGGGTGGCA	61.4	202
GnRH	GCAGATCCCTA AGAGGTGAA	CCGCTGTTGT TCTGTTGACT	55	201

### Statistical Analysis

Differences in gene expression examined *via* RT-PCR were analyzed by a one-way ANOVA followed by Tukey’s multiple comparison test for *post hoc* analysis (*p < 0.05, **p < 0.01, ***p < 0.001). Statistics were performed using the Prism software.

## Results

### Hypothalamic Neuropeptide and Receptor Expression Level in the Hypothalamus across the Reproductive Span

To characterize changes in the reproductive axis during the transition from reproductive competence to reproductive senescence in female rats, naturally aging rats that exhibited different stages of reproductive decline were utilized. Expression profiles in four groups were examined: a 3-month regularly cycling group labeled “young,” an 8-month regularly cycling group referred to as “middle-aged cycling,” a 10-month group of irregularly cycling rats referred to as “irregularly cycling,” and a 12-month group of acyclic animals referred to as “acyclic.”

**Figure 1:**
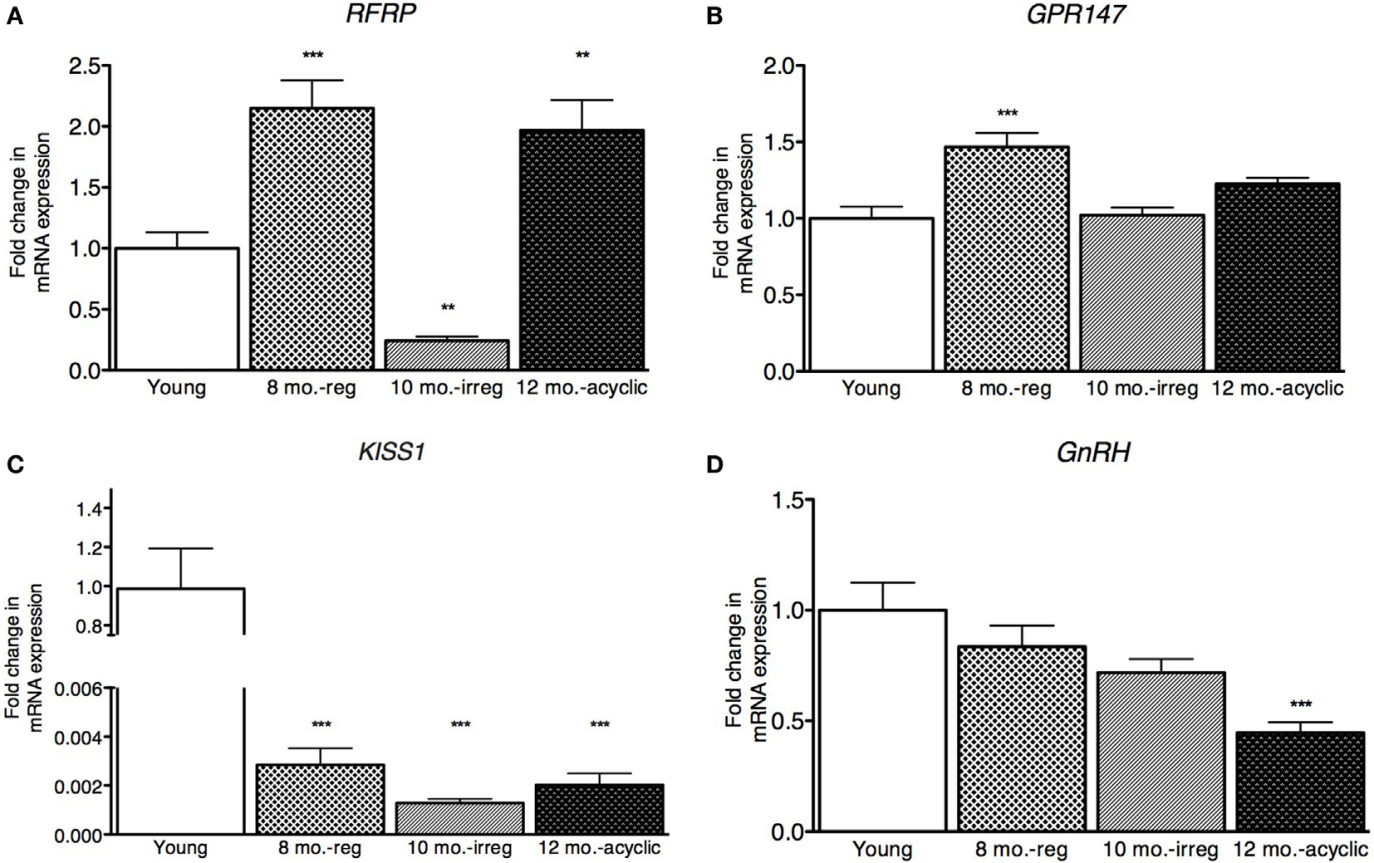
**Hypothalamic peptide mRNA expression in female rats**. **(A–D)** Gene expression changes in the hypothalamus at different age ranges through middle age. Young = 3-month regularly cycling females, 8-month-old reg. = 8-month-old females with regular estrous cycles, 10-month-old irreg. = 10-month-old females exhibiting irregular cycles, i.e., over 6–7 days rather than 4–5, and 12-month-old acyclic = 12-month-old rats that exhibit persistent estrous or diestrous over a period of more than 14 days. mRNA levels of all (mean ± SEM, *N* = 18/group for young and 8-month-old reg., *N* = 21 for 10-month-old irreg., and *N* = 12 for 12-month-old acyclic) were determined using qRT-PCR relative to the ribosomal reference gene RPLP. Estrous cycle staging was determined by inspection of daily vaginal smears (*p < 0.05, **p < 0.01, ***p < 0.001).

Hypothalamic *RFRP3* mRNA levels were significantly higher in middle-aged and acyclic females compared with young cycling females (relative fold change, 8 months old, 2.15 ± 0.23, 12 months old, 1.97 ± 0.25, Figure 1A). *RFRP3* was significantly lower in irregularly cycling females (10 months old, 0.24 ± 0.04, Figure 1A) compared with all other groups. Expression of the RFRP3 receptor, *GPR147*, was significantly higher in middle-aged animals regularly cycling females, relative to young controls (relative fold change, 1.5 ± 0.09, Figure 1B).

Kisspeptin is a potent activator of GnRH, and others have found that *KiSS1* decreases with aging (16). In our study, we also found a significant decrease in expression of *KiSS1* in all groups compared with young controls (relative fold change, 8 months old, 0.002 ± 1.9 × 10^−3^, 10 months old, 0.001 ± 1.8 × 10^−4^, and 12 months old, 0.002 ± 4.7 × 10^−4^, Figure 1C). We also see a downstream decrease in *GnRH* mRNA levels, as the acyclic animals exhibit a significant downregulation of GnRH compared with both the young controls and regularly cycling middle-aged animals (0.45 ± 0.05, Figure 1D).

### Pituitary Expression Level in the Pituitary across the Reproductive Span

**Figure 2:**
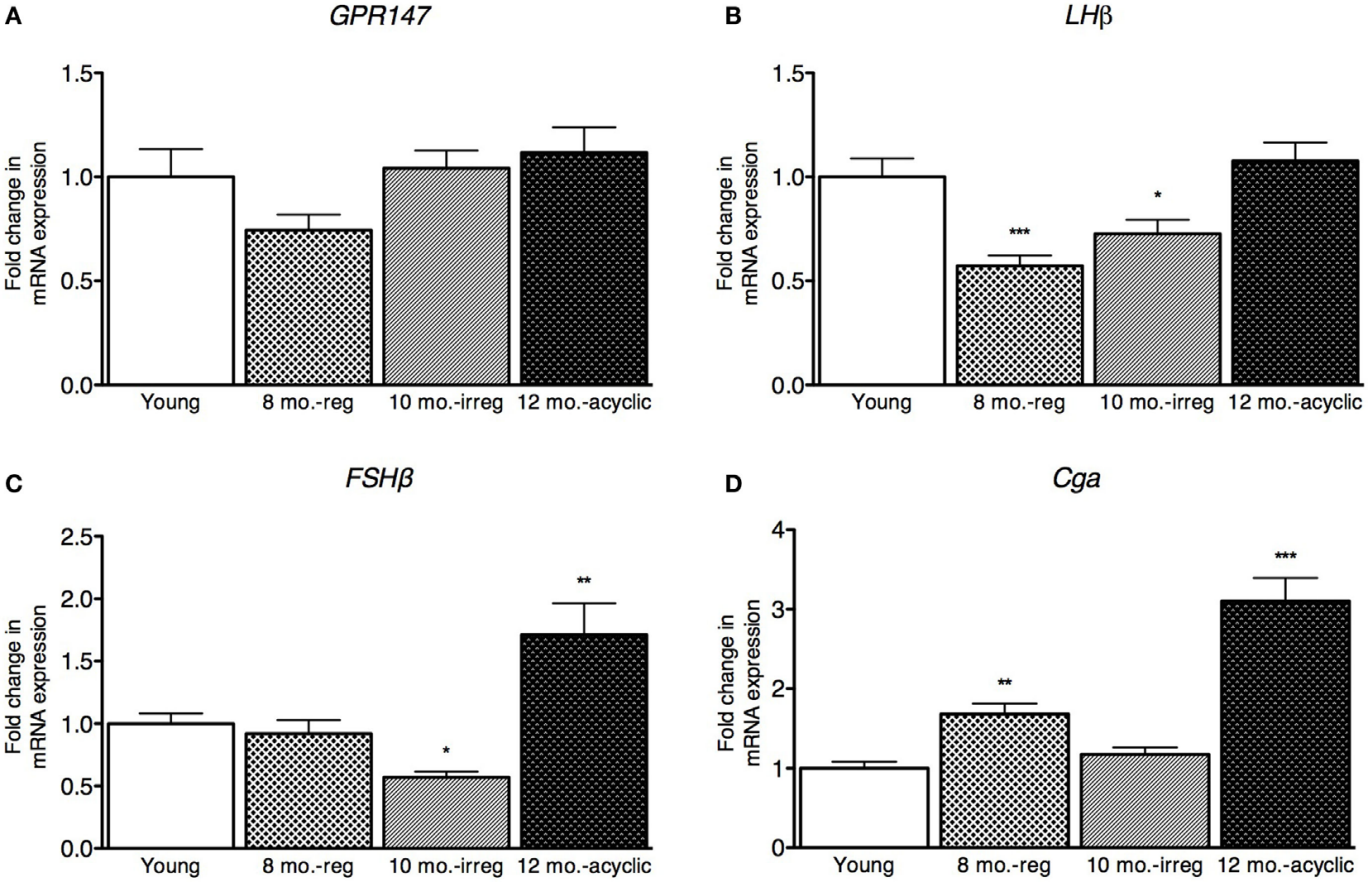
**Pituitary mRNA expression in female rats**. **(A–D)** Gene expression changes in the pituitary at different age ranges through middle age. Young = 3-month regularly cycling females, 8-month-old reg. = 8-month-old females with regular estrous cycles, 10-month-old irreg. = 10-month-old females exhibiting irregular cycles, i.e., over 6–7 days rather than 4–5, and 12-month-old acyclic = 12-month-old rats that exhibit persistent estrous or diestrous over a period of more than 14 days. mRNA levels of all (mean ± SEM, *N* = 18/group for young and 8-month-old reg., *N* = 21 for 10-month-old irreg., and *N* = 12 for 12-month-old acyclic) were determined using qRT-PCR relative to the ribosomal reference gene RPLP. Estrous cycle staging was determined by inspection of daily vaginal smears (*p < 0.05, **p < 0.01, ***p < 0.001).

Next, we examined gonadotropin changes in the pituitary and found no differences in expression of the RFRP3 receptor, *GPR147* across the four groups (Figure 2A). However, the expression level of the LH subunit β (LHβ) in the pituitary was decreased in the middle-aged regularly cycling group (relative fold-change, 0.57 ± 0.22, Figure 2B) and irregularly cycling animals (0.73 ± 0.31, Figure 2B) relative to young controls. In acyclic rats, LHβ mRNA expression was not significantly different from young controls (1.1 ± 0.31). FSHβ expression was decreased in irregularly cycling females (0.57 ± 0.21, Figure 2C) relative to young controls, but increased in acyclic females, compared with the young controls (1.71 ± 0.88). Synthesis of LH and FSH requires a common glycoprotein alpha subunit (Cga), in addition to a specific beta subunit. Cga expression was increased in the middle-aged group (1.68 ± 0.55, Figure 2D) relative to young and 10-month-old irregularly cycling females and the acyclic group (3.00 ± 1.00) relative to all other groups.

### RFRP3 and GPR147 Expression Increase Dramatically in the Aging Ovary

**Figure 3:**
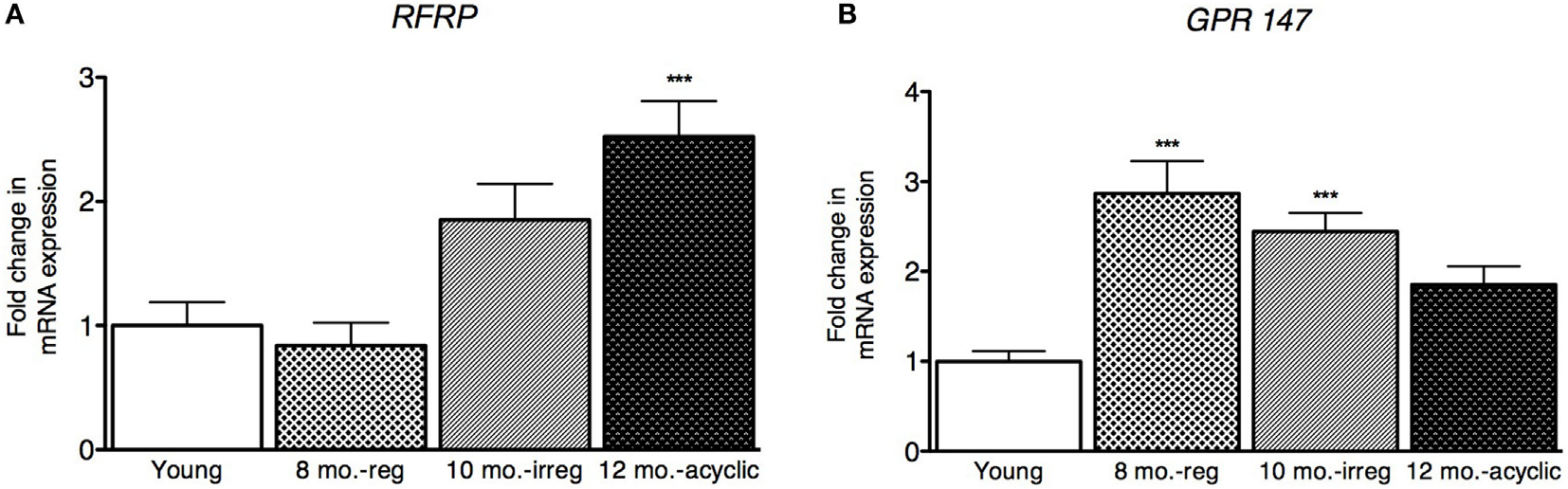
**Ovarian hormone mRNA levels in female rats**. **(A,B)** Gene expression changes in the ovary at different age ranges through middle age. Young = 3-month regularly cycling females, 8-month-old reg. = 8-month-old females with regular estrous cycles, 10-month-old irreg. = 10-month-old females exhibiting irregular cycles, i.e., over 6–7 days rather than 4–5, and 12-month-old acyclic = 12-month-old rats that exhibit persistent estrous or diestrous over a period of more than 14 days. mRNA levels of all (mean ± SEM, *N* = 18/group for young and 8-month-old reg., *N* = 21 for 10-month-old irreg., and *N* = 12 for 12-month-old acyclic) were determined using qRT-PCR relative to the ribosomal reference gene RPLP. Estrous cycle staging was determined by inspection of daily vaginal smears (*p < 0.05, **p < 0.01, ***p < 0.001).

Next, we explored how *RFRP3* and *GPR147* changed in the ovaries during reproductive decline. We found that *RFRP3* was higher in the ovaries in the acyclic group (2.5 ± 0.29, Figure 3A) compared with young and middle-aged cycling females. *GPR147* was upregulated in both middle-aged and irregularly cycling females compared with young animals (8 months old, 2.9 ± 0.34 and 10 months old, 2.44 ± 0.21, Figure 3B).

## Discussion

The present findings suggest that the inhibitory neuropeptide, RFRP3, may play a role in the initiation of reproductive decline in female rats, prior to change in cyclicity. Specifically, we found that hypothalamic RFRP3 expression increases significantly in middle age, concomitant with an increase in the RFRP3 receptor, *GPR147*, in the hypothalamus and gonads. These findings imply a transient increase in RFRP3 signaling around 8 months of age, prior to the onset of cycle irregularity. This transient increase in inhibitory signaling possibly acts as an initiating signal to inhibit regular cycling, leading to subsequent reproductive decline. This initial inhibitory signaling at 8 months is followed by a decrease in *KiSS1* in the hypothalamus. RFRP3 receptor mRNA has been shown to colocalize with KiSS1 neurons (19), suggesting that increased RFRP3 expression in middle age prior to reproductive cessation might act to signal *KiSS1*-expressing cells to further reproductive decline. Though transient, this effect may be sufficient to trigger a downstream decrease in HPG axis activity, as we see decreases in *LH*β and *FSH*β subunits in early aging. Since RFRP3 mRNA levels were measured in RNA extracted from whole hypothalamic in this study, it cannot provide nucleus-specific localization. However, RFRP3 expression was only documented in the DMH (12, 20). The data presented in this study provide a timeline of changes in expression of the major regulators, providing correlative evidence for the role played by RFRP3. Future studies using RFRP shRNA or overexpression viral vector or pharmacological inhibition of the RFRP3 receptor GPR147 would be required in order to prove the mechanistic role of RFRP3 in the timing of senescence. For example, a recently published inducible RFRP3 shRNA lentiviral vector (21) could be used to test the effect of blocking the transient increase in RFRP3 on onset of senescence. Alternatively, use of a GPR147 antagonist would provide information as to the role of RFRP3 in reproductive senescence, and the compound GJ14 might be a useful tool in this respect (22).

Previous studies have shown that LH serum concentrations decrease with aging, whereas FSH serum concentrations increase (6). This decrease in LH is likely triggered by a decrease in GnRH stimulation from the hypothalamus. It is hypothesized that an increase in FSH is seen in rodents, in contrast to what is observed in women, because follicles continue to develop throughout aging. However, these follicles are acyclic as they do not produce corpus lutea. The increase in *LH*β, *FSH*β, and *Cga* mRNA levels in acyclic, aged females has been seen in previous studies (23, 24) and is hypothesized to represent a decrease in the ability to transcribe gonadotropin proteins successfully, or potentially a decrease in the ability to release the gonadotropins. Here, we show that, similar to our hypothalamic findings, early decreases in LH and FSH transcription precede cycle cessation and increase thereafter, with this latter decrease likely representing the loss of negative feedback control.

Perhaps the most striking finding in this study is the increase in both *RFRP3* and *GPR147* in the ovaries as aging progresses. The increase in RFRP3 is maintained through at least 12 months of age female become acyclic. What is particularly interesting is the different kinetics of RFRP3 regulation in the hypothalamus and the gonads. RFRP3 increases transiently in the hypothalamus in middle-aged animals while still cycling normally, is suppressed in irregularly cycling females, and is upregulated again when cycling ceases. In contrast, RFRP3 expression in the ovaries increases after cycle irregularity begins and is maintained at least until cycles cease. This differential expression dynamics can potentially reflect hypothalamic RFRP signaling inducing subsequent downstream suppression of the axis. The correlative evidence described in this study would require further experimental manipulations to discover the mechanistic hierarchy. For instance, is the increase of ovarian RFRP3 induced by the hypothalamic RFRP3 increase, or occurs independently? There is evidence for local RFRP3 production in the ovary that might be independently regulated, separate from signals from the hypothalamus, providing support for this latter possibility (25–27). Decreased gonadal steroid production throughout the stages of reproductive decline (6) is thought to result from decreased preovulatory gonadotropin release. Our data suggest that this decrease in gonadal steroids may be due to local inhibition *via* RFRP3 in the ovaries.

Successful reproduction is dependent on the coordination of many endocrinological events in the brain and the periphery. In the aging rat, HPG axis functionality decreases, manifesting as decreases in GnRH synthesis and release, decreases in LH, and a decrease in gonadal steroids. However, the regulatory cues that initiate the transition from function to decline are largely yet unknown. We have described here the dynamic regulation of key neuropepetides in the HPG axis along this transition. Importantly, a transient increase in the expression of RFRP3 and its receptor precedes irregular cycling, and later cycling cessation, adding a new piece to the puzzle of reproductive decline.

## Author Contributions

AG, GB, LK, and DK designed experiments and wrote the manuscript. AG and SM performed experiments. AG analyzed data.

## Conflict of Interest Statement

The authors declare that the research was conducted in the absence of any commercial or financial relationships that could be construed as a potential conflict of interest.

## References

[1] Wise PM Smith MJ Dubal DB Wilson ME Rau SW Cashion AB Neuroendocrine modulation and repercussions of female reproductive aging. Recent Prog Horm Res (2002) 57:235–56. 10.1210/rp.57.1.235 12017546

[2] Hall JE. Neuroendocrine physiology of the early and late menopause. Endocrinol Metab Clin North Am (2004) 33(4):637–59. 10.1016/j.ecl.2004.08.002 15501638

[3] Hall JE. Neuroendocrine changes with reproductive aging in women. Semin Reprod Med (2007) 25(5):344–51. 10.1055/s-2007-984740 17710730

[4] Fitzgerald C Zimon AE Jones EE Aging and reproductive potential in women. Yale J Biol Med (1998) 71(5):367–81. 10527364 PMC2578931

[5] LeFevre J McClintock MK. Reproductive senescence in female rats: a longitudinal study of individual differences in estrous cycles and behavior. Biol Reprod (1988) 38(4):780–9. 10.1095/biolreprod38.4.780 3401536

[6] Lu KH Hopper BR Vargo TM Yen SSC Chronological changes in sex steroid, gonadotropin and prolactin secretion in aging female rats displaying different reproductive states. Biol Reprod (1979) 21(1):193–203. 10.1095/biolreprod21.1.193 573635

[7] Le WW Wise PM Murphy AZ Coolen LM Hoffman GE Parallel declines in Fos activation of the medial anteroventral periventricular nucleus and LHRH neurons in middle-aged rats. Endocrinology (2001) 142(11):4976–82. 10.1210/en.142.11.4976 11606466

[8] Rubin BS Lee CE King JC. A reduced proportion of luteinizing hormone (LH)-releasing hormone neurons express Fos protein during the preovulatory or steroid-induced LH surge in middle-aged rats. Biol Reprod (1994) 51(6):1264–72. 10.1095/biolreprod51.6.1264 7888504

[9] Zuo Z Mahesh VB Zamorano PL Brann DW Decreased gonadotropin-releasing hormone neurosecretory response to glutamate agonists in middle-aged female rats on proestrus afternoon: a possible role in reproductive aging? Endocrinology (2013) 137(6):2334–8. 10.1210/endo.137.6.8641183 8641183

[10] Kermath BA Gore AC. Neuroendocrine control of the transition to reproductive senescence: lessons learned from the female rodent model. Neuroendocrinology (2012) 96(1):1–12. 10.1159/000335994 22354218 PMC3574559

[11] Downs JL Wise PM. The role of the brain in female reproductive aging. Mol Cell Endocrinol (2009) 299(1):32–8. 10.1016/j.mce.2008.11.012 19063938 PMC2692385

[12] Kriegsfeld LJ Gibson EM Williams WP III Zhao S Mason AO Bentley GE The roles of RFamide-related peptide-3 in mammalian reproductive function and behaviour. J Neuroendocrinol (2010) 22(7):692–700. 10.1111/j.1365-2826.2010.02031.x 20646173 PMC2908924

[13] Ukena K Iwakoshi E Minakata H Tsutsui K. A novel rat hypothalamic RFamide-related peptide identified by immunoaffinity chromatography and mass spectrometry. FEBS Lett (2002) 512(1–3):255–8. 10.1016/S0014-5793(02)02275-5 11852091

[14] Ukena K Tsutsui K. Distribution of novel RFamide-related peptide-like immunoreactivity in the mouse central nervous system. Neurosci Lett (2001) 300(3):153–6. 10.1016/S0304-3940(01)01583-X 11226634

[15] Neal-Perry G Lebesgue D Lederman M Shu J Zeevalk GD Etgen AM. The excitatory peptide kisspeptin restores the luteinizing hormone surge and modulates amino acid neurotransmission in the medial preoptic area of middle-aged rats. Endocrinology (2009) 150(8):3699–708. 10.1210/en.2008-1667 19423763 PMC2717872

[16] Lederman MA Lebesgue D Gonzalez VV Shu J Merhi ZO Etgen AM Age-related LH surge dysfunction correlates with reduced responsiveness of hypothalamic anteroventral periventricular nucleus kisspeptin neurons to estradiol positive feedback in middle-aged rats. Neuropharmacology (2010) 58(1):314–20. 10.1016/j.neuropharm.2009.06.015 19559035 PMC2901500

[17] Zhao S Fernald RD. Comprehensive algorithm for quantitative real-time polymerase chain reaction. J Comput Biol (2005) 12(8):1047–64. 10.1089/cmb.2005.12.1047 16241897 PMC2716216

[18] Pfaffl MW. A new mathematical model for relative quantification in real-time RT-PCR. Nucleic Acids Res (2001) 29(9):e45. 10.1093/nar/29.9.e45 11328886 PMC55695

[19] Rizwan MZ Poling MC Corr M Cornes PA Augustine RA Quennell JH RFamide-related peptide-3 receptor gene expression in GnRH and kisspeptin neurons and GnRH-dependent mechanism of action. Endocrinology (2012) 153(8):3770–9. 10.1210/en.2012-1133 22691552

[20] Kriegsfeld LJ Mei DF Bentley GE Ubuka T Mason AO Inoue K Identification and characterization of a gonadotropin-inhibitory system in the brains of mammals. Proc Natl Acad Sci U S A (2006) 103(7):2410. 10.1073/pnas.0511003103 16467147 PMC1413747

[21] Geraghty AC Muroy SE Zhao S Bentley GE Kriegsfeld LJ Kaufer D. Knockdown of hypothalamic RFRP3 prevents chronic stress-induced infertility and embryo resorption. Elife (2015) 4:e04316. 10.7554/eLife.04316 25581095 PMC4289855

[22] Kim JS Brownjohn PW Dyer BS Beltramo M Walker CS Hay DL Anxiogenic and stressor effects of the hypothalamic neuropeptide RFRP-3 are overcome by the NPFFR antagonist GJ14. Endocrinology (2015) 156(11):4152–62. 10.1210/en.2015-1532 26259035

[23] Chen H. Gene expression by the anterior pituitary gland: effects of age and caloric restriction. Mol Cell Endocrinol (2004) 222(1–2):21–31. 10.1016/j.mce.2004.05.004 15249122

[24] Gruenewald DA Naai MA Hess DL Matsumoto AM. The Brown Norway rat as a model of male reproductive aging: evidence for both primary and secondary testicular failure. J Gerontol (1994) 49(2):B42–50. 10.1093/geronj/49.2.B42 8126345

[25] Oishi H Klausen C Bentley GE Osugi T Tsutsui K Gilks CB The human gonadotropin-inhibitory hormone ortholog RFamide-related peptide-3 suppresses gonadotropin-induced progesterone production in human granulosa cells. Endocrinology (2012) 153(7):3435–45. 10.1210/en.2012-1066 22691551

[26] Singh P Krishna A Sridaran R Tsutsui K. Immunohistochemical localization of GnRH and RFamide-related peptide-3 in the ovaries of mice during the estrous cycle. J Mol Histol (2011) 42(5):371–81. 10.1007/s10735-011-9340-8 21769536 PMC3346712

[27] Singh P Krishna A Sridaran R Tsutsui K. Changes in GnRH I, bradykinin and their receptors and GnIH in the ovary of *Calotes versicolor* during reproductive cycle. Gen Comp Endocrinol (2008) 159(2–3):158–69. 10.1016/j.ygcen.2008.08.016 18809405 PMC7927428

